# Construction of Novel Chloroplast Expression Vector and Development of an Efficient Transformation System for the Diatom *Phaeodactylum tricornutum*

**DOI:** 10.1007/s10126-014-9570-3

**Published:** 2014-04-26

**Authors:** Wei-Hong Xie, Cong-Cong Zhu, Nai-Sheng Zhang, Da-Wei Li, Wei-Dong Yang, Jie-Sheng Liu, Ramalingam Sathishkumar, Hong-Ye Li

**Affiliations:** 1 Key Laboratory of Eutrophication and Red Tide Prevention of Guangdong Higher Education Institute, Jinan University, 510632 Guangzhou, China; 2 Plant Genetic Engineering Laboratory, Department of Biotechnology, Bharathiar University, Coimbatore, Tamil Nadu 641046 India

**Keywords:** Bioreactor, Plastid transformation, Microalga, Diatom, *Phaeodactylum tricornutum*

## Abstract

Plastids are ideal subcellular hosts for the expression of transgenes and have been successfully used for the production of different biopolymers, therapeutic proteins and industrial enzymes. *Phaeodactylum tricornutum* is a widely used aquatic feed species. In this study, we focused on developing a high-efficiency plastid expression system for *P. tricornutum*. In the plastid transformation vector, the site selected for integration was the transcriptionally active intergenic region present between the *trnI* and *trnA* genes, located in the IR (inverted repeat) regions of the plastid genome. Initially, a *CAT* reporter gene (encoding chloramphenicol acetyltransferase) was integrated at this site in the plastid genome. The expression of *CAT* in the transformed microalgae conferred resistance to the antibiotic chloramphenicol, which enabled growth in the selection media. Overall, the plastid transformation efficiency was found to be approximately one transplastomic colony per 1,000 microalgae cells. Subsequently, a heterologous gene expression cassette for high-level expression of the target gene was created and cloned between the homologous recombination elements. A TA cloning strategy based on the designed *Xcm*I-*Xcm*I sites could conveniently clone the heterologous gene. An *eGFP* (green fluorescent protein) reporter gene was used to test the expression level in the plastid system. The relatively high-level expression of eGFP without codon optimisation in stably transformed microalgae was determined to account for 0.12 % of the total soluble protein. Thus, this study presents the first and convenient plastid gene expression system for diatoms and represents an interesting tool to study diatom plastids.

**Electronic supplementary material:**

The online version of this article (doi:10.1007/s10126-014-9570-3) contains supplementary material, which is available to authorized users.

## Introduction

Marine diatoms are responsible for a high level of productivity and are required to sustain life in the sea. *Phaeodactylum tricornutum* is an essential phytoplankton used as a live feed in aquaculture, as it is rich in protein and fatty acid contents. *P. tricornutum* is also rich in EPA (eicosapentaenoic fatty acid), a ω-3 fatty acid that is a valuable nutritional supplement. *P. tricornutum* is one of the four diatoms for which the genome sequence is available, with the rest three being *Thalassiosira pseudonana*, *Fragilariopsis cylindrus* and *Pseudo-nitzschia multiseries* (http://genome.jgi-psf.org). These attributes make *P. tricornutum* a promising candidate for genetic modification.

In *P. tricornutum*, a stable nuclear transformation method is the only previously reported method for transformation (Niu et al. 2012; Apt et al. 1996b; Miyagawa et al. 2009). Plastids can be an ideal site of storage for the recombinant proteins compared to the cytoplasm, as adverse effects due to over accumulation can be avoided (Bogorad 2000). Plastid expression systems have been used successfully for the production of different biopolymers, therapeutic proteins and industrial enzymes. In plant cells, the presence of many transgene copies of the plastid genome per cell increases the accumulation of the foreign protein several folds, although the amount of the recombinant protein is mainly determined by factors such as transcriptional and posttranscriptional regulation of foreign genes, which determine the protein stability (De Marchis et al. 2012). For example, human somatotropin has been reported to accumulate at levels of up to 7 % of the total soluble protein (TSP) when expressed in the plastid, which is 300-fold greater than that of nuclear transformants (Staub et al. 2000). Furthermore, human serum albumin accumulated at levels up to 11.1 % of the TSP, which is 500-fold higher than that of nuclear transformants (Millán et al. 2003). Additionally, plastid transformation has several advantages over nuclear transformation. For example, gene integration in the plastome occurs through homologous recombination, thus avoiding position effects; the high copy number of plastomes per cell and the lack of silencing machinery allow high and stable gene expression (Staub et al. 2000; McBride et al. 1995; Daniell et al. 1998; Sidorov et al. 1999). However, the success of plastid transformation largely depends on the species. Reproducible protocols are available for plastid transformation for some plant species (Maliga and Bock 2011). So far, there have been only few reports on transgene expression in the plastids of microalga, such as the green alga *Chlamydomonas reinhardtii* (Muto et al. 2009), the red alga *Porphyridium* sp. (Lapidot et al. 2002) and the euglenoid *Euglena gracilis* (Doetsch et al. 2001). Recently, a unique immunotoxin for cancer treatment has been successfully produced in *Chlamydomonas* plastids as a soluble protein with enzymatic activity, demonstrating a novel approach to the control of the disease (Tran et al. 2013). As the plastid genome of *P. tricornutum* was sequenced (Oudot-Le Secq et al. 2007), genetic manipulation of the plastid genome has attracted much attention. Genome mutations have been induced in the *psbA* gene in *P. tricornutum* plastids and consequently regulated photosynthesis (Materna et al. 2009). Here, we report the construction of a plastid expression vector and transformation system for overexpression of foreign genes in *P. tricornutum* plastids. Analysis of the plastid genome sequence of *P. tricornutum* revealed that it contains inverted repeat (IR) regions, distinct from the well-known microalgae *Chlorella* sp. in which there is no IR region (Wakasugi et al. 1997). In this study, using the available plastid genome sequence for *P. tricornutum*, we have developed a high-efficiency plastid transformation system by introducing a specially designed TA cassette for cloning a heterologous gene between the two homologous recombination fragments *trnA* and *trnI* from the IR regions of the plastid vector.

## Materials and Methods

### Microalga Material Culture Conditions

*P. tricornutum* was obtained from the Freshwater Algae Culture Collection, Institute of Hydrobiology, China (Cat. No. FACHB-863). The microalgae were grown as batch cultures in Erlenmeyer flasks containing f/2 medium, which was sterilised through 0.22-μm filters (Millipore, Billerica, MA, USA). The cultures were grown at 21 ± 1 °C in an artificial climate incubator. Cool-white fluorescent tubes provided an irradiance of 200 μmol photons m^−2^ s^−1^ under long-day light conditions (15/9 h light/dark regime). In the PCR reactions, *Pfu* DNA polymerase was used to reduce the likelihood of introducing DNA mutations.

### Plasmid Construction for Plastid Transformation

**Fig. 1:**
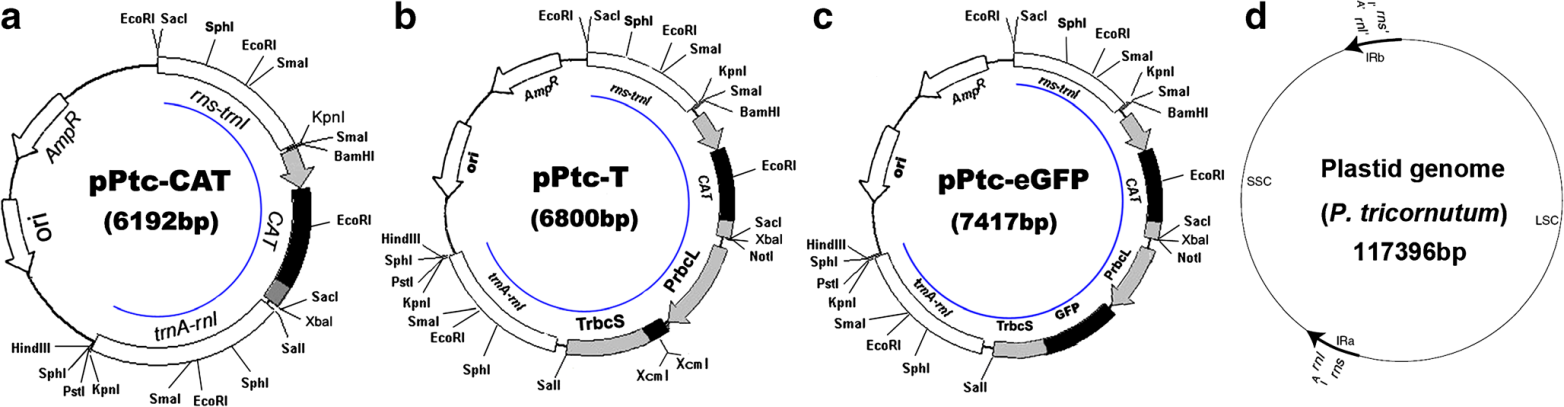
Schematic maps showing plasmid constructs. **a** pPtc-CAT, **b** pPtc-T, **c** pPtc-eGFP and **d** map of plastid genome showing the integration sites. Annotations of the plasmid maps are given below: *rns*-*trnI* and *trnA*-*rnl*: The flanking regions derived from the *P. tricornutum* plastid genome used for homologous recombination during plastid transformation, *CAT*: reporter gene cassette encoding chloramphenicol acetyltransferase, P*rbcL*: promoter of the rubisco large subunit gene from *P. tricornutum* and T*rbcS*: terminator of the rubisco small subunit gene from *P. tricornutum*; the *CAT* expression cassette was derived from *Escherichia coli*; *eGFP*: reporter gene encoding green fluorescent protein and Amp^R^: ampicillin resistance gene cassette from the plasmid pMD19. The *arcs* inside the vectors indicate the fragments recombining within the plastid genome. The map of plastid genome was derived from Oudot-Le Secq et al. (2007), where “I” and “A” indicate *trnI* and *trnA*, respectively

The plasmid pPtc-CAT (Fig. 1a), a plastid transformation vector, was constructed for testing the integration and expression of the *CAT* reporter gene in the plastid genome. It was generated by cloning the homologous recombination elements *trnA/trnI* and the *CAT* expression cassette into pMD19 (TaKaRa, Dalian, China). The *CAT* expression cassette was derived from a bacterial source. Chloramphenicol acetyltransferase (*CAT*) is a bacterial enzyme that detoxifies the antibiotic chloramphenicol, and it is responsible for chloramphenicol resistance. First, the total DNA from *P. tricornutum* was extracted using a Universal Genomic DNA Extraction Kit Ver.3.0 (TaKaRa, Dalian, China). Regions encompassing 1.1-kb of *rns-trnI* and 1.3-kb of *trnA-rnl* in the *P. tricornutum* plastid genome were selected because they include homologous recombination elements. The fragments *trnA-rnl* and *rns-trnI* were cloned by PCR using total DNA as the template and primers, including P1 (5′-CGAGCTCCGAGCTCACTGGGCGTAAAGCGTCTGT-3′ *Sac*I site is underlined), P2 (5′-GGGGTACCGGGGTACCTTGGGCCATTCTGGATTTG-3′, *Kpn*I site is underlined), P3 (5′-ACGCGTCGACAACTGCAGCGGGGGTATAGCTCAGTTGG-3′, *Sal*I site is underlined) and P4 (5′-AACTGCAGACATGCATGCTTTCGTTACTCAAGCCGACATT-3′, *Pst*I site is underlined). The *Sac*I and *Kpn*I restriction sites were added at the two ends of *rns-trnI* fragment for subcloning into the same sites in pMD19. Subsequently, the *Sal*I and *Pst*I sites flanking the *trnA-rnl* fragment were cloned using the same sites. Finally, the *CAT* expression cassette was cloned by PCR using the plasmid pLysS (Novagen, USA) as a template with the following primers: P5 (5′-CGGGATCCCGGGATCCAGCATCACCCGACGCACT-3′, the *Bam*HI site is underlined) and P6 (5′-GCTCTAGAGCTCTAGATAACGACCCTGCCCTGAAC-3′, the *Xba*I site is underlined). The *Bam*HI and *Xba*I sites flanking the *CAT* expression cassette were cloned into the above plasmid harbouring the fragments *trnA-rnl* and *rns-trnI*, leading to the plastid transformation vector, pPtc-CAT.

To achieve a high-efficiency plastid vector for the cloning of target genes, the TA-cloning plasmid pPtc-T (Fig. 1b) was constructed. Initially, a target gene expression cassette of P*rbcL*-*Xcm*I-*Xcm*I-T*rbcS* was generated through the ligation-independent cloning (LIC) method. The P*rbcL* and T*rbcS* sequences are the promoter for the large subunit (*rbcL*) and the terminator of the small subunit (*rbcS*) of the rubisco genes, respectively. The 240-bp P*rbcL* fragment was amplified by PCR using total DNA as a template with the forward primer Pt358 (5′-gcTCTAGAGCGGCCGCAAACTTCTAAAACTTTCAATTAAAAGCTATTCT-3′ the *Xba*I-*Not*I sites are underlined) and the reverse primer Pt347 (5′-TGATTTCTCCTTGGAATAAAAAGGCAATAT-3′). The PCR product was subsequently amplified by PCR with the forward primer Pt358 and the overlapped reverse primers Pt360a (5′-TCCTGGCCACCACAGGTGTGGTGATTTCTCCTTGGAATAAAAAGG-3′ the *Xcm*I site is underlined) and Pt360b (5′-GTTTTTGTTCACCACCACTCTCCTGGCCACCACAGGTG-3′, the *Xcm*I site is underlined). Thus the two *Xcm*I sites were added to the 3′ end of P*rbcL*. The 260-bp T*rbcS* fragment was cloned by PCR using total DNA as a template with the forward primer Pt344 (5′-TTTTTAGTAACAAAATAAAATTAAAAAATGTTAAT-3′) and the reverse primer Pt359 (5′-gcGTCGACCATTTTTTGTCATTGTGATAAGCTAAAGT-3′, the *Sal*I site is underlined). The PCR product was subsequently amplified by PCR with the forward primer Pt357Myc (5′-GAACAAAAACTCAGTGAAGAAGATCTTTAATTTTTAGTAACAAAATAAAATTAAA-3′) and the reverse primer Pt359. Thus, the Myc-tagged sequence (encoding amino acids EQKLISEEDL) and stop codon were added upstream of T*rbcS*. The fragments P*rbcL*-*Xcm*I-*Xcm*I and Myc-T*rbcS* were ligated by LIC. The resulting fragment and plasmid pPtc-CAT were digested with *Sal*I and *Xba*I and then ligated to a new vector pPtc-T for plastid transformation.

The gene of interest can be easily cloned into the vector pPtc-T using the TA cloning strategy. To generate the extra “T” at either 3′-end of the linearised vector, it was predigested with *Xcm*I. To test the vector pPtc-T for target gene cloning and expression in the plastid, the *eGFP* (green fluorescent protein) reporter gene was cloned between P*rbcL* and T*rbcS* in the pPtc-T by TA cloning, generating the vector pPtc-eGFP (Fig. 1c). The *eGFP* coding region was amplified with Taq polymerase to add an additional “A” at the 3′-end using the primers eGFPf (5′-ACC ATGGTGAG CAAGGGCGAG GAGCTG-3′) and eGFPr (5′-AACTCGAGCTTGTACAGCTCGTC-3′). Hence, the vector pPtc-eGFP utilises the plastid genomic sequences spanning the *trnA*-*trnI* region to target the heterologous gene into the plastid genome by homologous recombination (Fig. 1d).

### Transformation of *P. tricornutum* by Electroporation

Plastid transformation was carried out by electroporation of *P. tricornutum* using a Bio-Rad Gene Pulser Xcell apparatus (Niu et al. 2012). Microalgae were aseptically spread on f/2 medium, supplemented with agar (5 g/l) and chloramphenicol (30 g/l). Microalgal cells in the exponential phase were collected by centrifuging at 1,350*g* for 10 min. The pellets containing 0.5 × 10^7^ microalgal cells were resuspended in 150 μl 1.0 M NaCl then mixed with 150 μl 0.1 M mannitol and incubated in ice for 30 min. Aliquots of 0.4 ml were mixed with 0.4 μg of the plasmid pPtc-eGFP and transferred into an electroporation cuvette (Gene Pulser/MicroPulser Cuvette, 0.4 cm gap, Bio-Rad). After electroporation, the cells were transferred to 10 ml f/2 medium and incubated in the dark for 2 h, followed by incubation for 24 h (12 L:12 D). The cells were then collected by centrifugation at 1,500*g* for 5 min and resuspended in 1 ml medium. The cells were finally spread on f/2 selection medium supplemented with 5 g/l agar containing various concentrations of chloramphenicol (200, 250 and 300 mg/l).

### Analysis of the Transformed Microalgae

After 4 weeks of incubation of the plates under standard growth conditions, the putative transformed colonies were counted. The viable colonies were picked and inoculated into the fresh liquid f/2 medium containing 200 mg/l chloramphenicol and then subjected to five more subculture cycles on selection medium to obtain homoplastomic microalgae. To verify the plastid-transformed microalgae, the plastid DNA was extracted and used in PCR as a template. The plastids of *P. tricornutum* were isolated by sucrose gradient centrifugation. Cells in 50 ml culture in the exponential phase were harvested by centrifugation at 5,000*g* for 10 min at 4 °C. The algal pellet was ground to a fine powder in liquid nitrogen and transferred to 1.5 ml grinding buffer (0.3 M sucrose, 40 mM Tris–HCl (pH 7.8), 5 mM MgCl_2_, 1 mM PMSF) followed by incubation on ice for 5 min. The homogenate was subsequently separated by centrifugation at 350*g* for 10 min at 4 °C, and the pellet contained the nuclear fraction. The supernatant was carefully transferred to a new tube without disturbing the pellet, followed by centrifugation at 12,000*g* for 20 min at 4 °C, and the resultant pellet predominantly consisted of plastids. The plastid-rich fraction was confirmed by observation of chlorophyll fluorescence under a fluorescence microscope. The plastid DNA was extracted from the plastid-rich fraction using the Universal Genomic DNA Extraction Kit Ver.3.0 (TaKaRa).

To confirm the presence of the C*AT* gene in the microalgae, the cells were tested using PCR with the *CAT*-specific primers P5 and P6. Simultaneously, to validate the DNA integration and homologous recombination between *trnA* and *trnI* in the plastid genome in the transformed cells, PCR was performed with the primers IRf1 (5′-ATCGGCTAACTCCGTGCCAG-3′) or IRf2 (5′-TAACTATTTCTTATGACCTTTCC-3′) and CATr (5′-AGCAACTGACTGAAATGCCTC-3′). The primer IRf1 and IRf2 were designed in the region of the 5′ upstream of the *rns-trnI*, which were not included in the cloned *rns-trnI* elements. The primer CATr was derived from the *CAT* coding region. The PCR reaction mixture (25 μl) consisted of 250 ng template, 5 pmol of each primer, 0.75 U LA Taq polymerase (TaKaRa), 2.5 μl 10× PCR reaction buffer, 1.5 μl of 25 mM MgCl_2_ and 0.25 μl of each 10 mM dNTP. PCR amplification was initiated with denaturation at 95 °C for 3 min, followed by 35 cycles of 95 °C 1 min, 58 °C 1 min and 72 °C 1 min. The expected PCR band on the gel was purified and analysed by sequencing.

Southern blot analysis was carried out to further confirm the gene integration as described previously (Manuell et al. 2007) by using the DIG DNA Labeling and Detection Kit (Roche, USA) following the manufacturer’s instructions. The Universal Genomic DNA Extraction Kit Ver. 3.0 (TaKaRa) was used to extract the genomic DNA from the pPtc-eGFP transformed and untransformed control cells. Samples containing 5 μg of genomic DNA were digested with *Eco*RI. The *Eco*RI/*Xba*I fragment in the coding region of *CAT* (Fig. 1) was used to prepare a DIG-labelled probe.

### Transcription Levels of the *CAT* and *eGFP* Reporter Genes Quantified by Real-Time PCR

For quantification of the transcripts, the RNeasy Plant Mini kit (QIAGEN, USA) was used to extract the total RNA from 1 × 10^8^ pPtc-eGFP transformed and untransformed *P. tricornutum* cells. During extraction, the RNA samples were treated with QIAGEN RNase-free DNase I to remove residual-contaminating DNA. In total, 2 μg of total RNA was reverse transcribed with random hexamer primers using an Omniscript reverse transcription kit (QIAGEN). The RNA sample was used as template for cloning genes, while genes containing introns could be used as controls demonstrating complete DNA degradation. The primers for *CAT*, *eGFP* and *β-actin* housekeeping marker were designed according to previous reports (De Riso et al. 2009; Niu et al. 2012). The qPCR was conducted in 96-well optical reaction plates using a SYBR Green Kit (Ruizhen Co., China) according to the manufacturer’s instructions. Each sample was amplified in triplicate on a 7300 Sequence Detection System (Applied Biosystems, USA). The threshold cycle for each well was measured, and the relative transcription levels of the target gene compared with the control were quantified after normalisation to *β-actin*.

### Protein Expression Determined by Western Blot and ELISA Analysis

The expression of the target gene could be rapidly tested through the Myc-tagged polypeptide present in the pPtc-T vector. To examine eGFP expression, a protein extraction kit (KeyGEN, Nanjin, China) was used to extract the total protein from 1 × 10^8^ *P. tricornutum* cells transformed with pPtc-eGFP and an untransformed control. The protein concentrations were measured by the bicinchoninic acid assay (BCA assay). Twenty micrograms of protein from each sample was resolved by 12 % SDS–PAGE at 100 V for 100 min and stained with Coomassie Brilliant Blue G-250 to visualise the protein bands. Two other identical gels were electrotransferred to a PVDF membrane. The membranes were blocked in phosphate-buffered saline and 0.1 % Tween 20 (PBST) (137 mM NaCl, 2.7 mM KCl, 10 mM Na_2_HPO_4_, 1.8 mM KH_2_PO_4_, 0.5 % Tween 20, pH 7.6) containing 5 % bovine serum albumin (BSA) for 2 h at room temperature. After washing three times in PBST, the membranes were incubated with an anti-Myc antibody (Invitrogen, USA) or an anti-GFP antibody (Epitomics, USA) at a 1:5,000 dilution in 5 % BSA in PBST for 2 h at room temperature. The anti-GAPDH and anti-β-actin antibodies (Abcam, UK) were used as internal controls at a dilution of 1:5,000. The membranes were then washed three times with PBST and incubated with a horseradish peroxidase (HRP)-conjugated secondary antibody (Kangwei, China) at a 1:5,000 dilution in PBST for 1 h at room temperature. The blots were washed three times and developed with tetramethyl-benzidine (TMB) reagent (Beyotime, China).

To quantify the algae-expressed recombinant eGFP protein, ELISA was conducted using an ELISA Kit (TSZ Elisa, Fuxing Bio., Shanghai, China). Briefly, an anti-GFP antibody was used to coat the microtiter plates. Then, the algal total soluble protein samples or GFP standards and a HRP-conjugated secondary antibody were added, incubated and washed thoroughly. TMB substrate was added for the colour development, and the reaction was stopped using sulphuric acid. The eGFP was quantified according to the absorbance readings at OD450 using an ELISA reader (ELISA3000, DRG International Inc., NJ, USA) and the calculation from the eGFP standard curve.

### Laser-Scanning Confocal Microscopy

A Zeiss LSM 510 Meta inverted confocal laser-scanning microscope (Zeiss, Jena, Germany) was used for the analysis of eGFP localisation as described by Li et al. (2008), using cells in the stationary phase. By means of multitracking, eGFP was excited at 488 nm and detected via a 505–530-nm band pass filter; chlorophyll fluorescence was excited at 543 nm and detected with a 600-nm long-pass filter. The images were processed using the LSM 510 software (Zeiss, Jena, Germany).

### eGFP Expression Assayed by Flow Cytometry

eGFP expression in the transformed cells was quantified by flow cytometry, which is considered a sensitive method (Ducrest et al. 2002). Cells (1 × 10^4^) in the stationary phase were used for the eGFP fluorescence intensity assay. The cells were analysed using a FACS-Aria microflow cytometer (Becton Dickinson, NJ, USA). The dead cells and debris were eliminated using forward and side scatter parameters in the analysis. eGFP was excited at a wavelength of 488 nm, and the fluorescence was detected with a 530/15-nm bandpass filter in the FL1 channel.

## Results

### Construction of Plastid Transformation Vectors

Schematic maps of the plastid transformation vectors pPtc-CAT (Fig. 1a), pPtc-T (Fig. 1b) and pPtc-eGFP (Fig. 1c) are shown in Fig. 1. The homologous recombination elements *rns*-*trnI* and *trnA*-*rnl* from the *P. tricornutum* plastid genome were cloned into the plasmids. *CAT* and *eGFP* expression cassettes were inserted between the recombination elements. *P. tricornutum* was previously reported to be sensitive to chloramphenicol (Apt et al. 1996a, b), and we found that it could not survive ≥200 mg/l chloramphenicol treatment. Therefore, chloramphenicol was used as the selection marker for the expression vectors. The resultant plasmids pPtc-CAT, pPtc-T and pPtc-eGFP were 6,192, 6,800 and 7,417 bp, respectively. All the vectors were confirmed by restriction enzyme analysis and sequencing.

### Selection and Chloramphenicol Resistance of Plastid-Transformed Microalgae

**Fig. 2:**
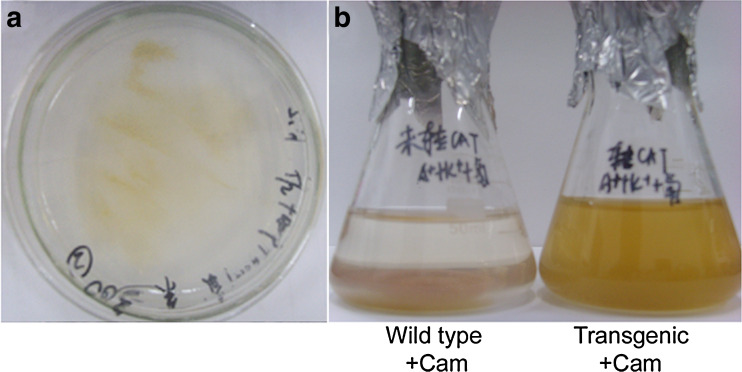
Plastid transformed microalgae. **a** Transformed cells expressing *CAT* were determined on plates supplemented with 300 mg/l chloramphenicol; **b** surviving cells were picked from the plate and grown in liquid medium supplemented with 300 mg/l chloramphenicol (*right flask*), while wild-type cells could not grow in medium with 200 mg/l chloramphenicol (*left flask*)

Transformed microalgae cells were selected on solid selection medium after electroporation, and putative transformed cells were streaked to purify and then cultured in liquid medium (Fig. 2). Transformed cells containing the plasmid pPtc-eGFP-expressing *CAT* grew well on the selection plates supplemented with 300 mg/l chloramphenicol (Fig. 2a), and these cells also grew well in the liquid medium supplemented with 300 mg/l chloramphenicol (Fig. 2b, right flask), whereas wild-type cells did not grow in the medium supplemented with 200 mg/l chloramphenicol (Fig. 2b, left flask). These results demonstrated that the heterologous *CAT* gene was successfully expressed in the microalgae, and it exhibited antibiotic resistance. The overall efficiency of plastid transformation was estimated to be approximately one transplastomic colony per 1,000 microalgae cells (5 × 10^3^ transformants in 5 × 10^6^ cells per 0.4 μg DNA), which is 100-fold higher than that obtained with the *P. tricornutum* nuclear transformation system (Miyagawa et al. 2009; Apt et al. 1996a, b), indicating the occurrence of high-efficiency homologous recombination in the plastid genome.

### Culture and Analysis of Plastid-Transformed Microalgae

**Fig. 3:**
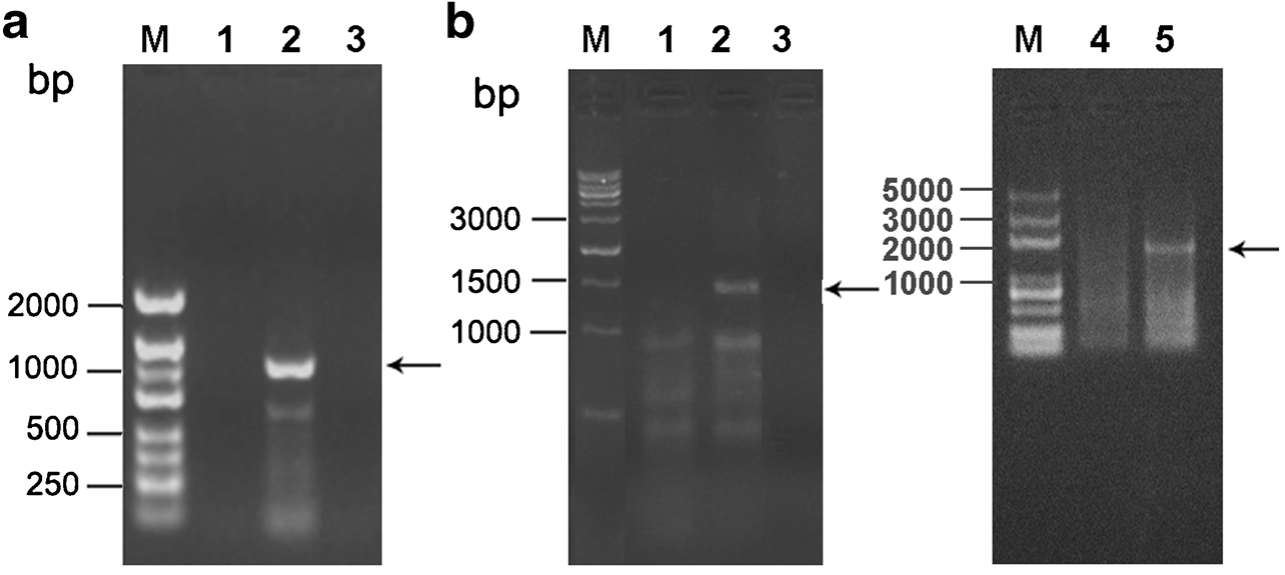
Heterologous gene integration in plastid-transformed microalgae analysed by PCR. **a** Analysis of the *CAT* cassette using the primers P5 and P6 and **b** analysis of homologous recombination region using the primer pairs of IRf1 + CATr (*left panel*) and IRf2 + CATr (*right panel*). *Lane M* molecular mass markers, *lane 1* PCR amplification of wild-type microalgae, *lane 2* PCR amplification of transformed microalgae, *lane 3* the negative control of the PCR amplification without a DNA template, *lane 4* PCR amplification of wild-type microalgae and *lane 5* PCR amplification of transformed microalgae; the *arrows* indicate the expected 1.1-kb CAT band, 1.49-kb IRf1 + CATr and 2.0-kb IRf2 + CATr homologous recombination region, respectively

**Fig. S1:**
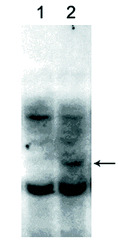
Southern blot analysis of heterologous gene integration in plastid-transformed microalgae. Lane 1, wild type; Lane 2, transformed line. Genomic DNA was digested with EcoRI and hybridised with a CAT coding region probe (left panel) or a genomic flanking probe (right panel). The arrow indicates the specific hybridisation band present in the transplastomic microalgae which was absent in wild type. (GIF 11 kb)

Transformed microalgae cultured in liquid medium were subjected to PCR analysis using the *CAT* cassette primers P5 and P6 and another primer set including IRf and CATr. Gel electrophoresis showed a 1.1-kb band as expected from the PCR with the P5 and P6 primers in the transformed microalgae (Fig. 3a, lane 2), but this band was absent in the wild type (Fig. 3a, lane 1). As expected, the 1.49 and 2.0-kb bands were obtained in the PCR with the IRf1 + CATr and IRf2 + CATr primers, respectively, in the transformed microalgae (Fig. 3b, lanes 2 and 5), but not in the wild type (Fig. 3b, lanes 1 and 4). The expected bands were purified and analysed by sequencing and showed the exact sequence including the rns-trnl on the transformation vector and the region on the plastid genome, indicating the integration of the *CAT* cassette in the plastid genome of the microalgae by homologous recombination. The gene integration was further confirmed by Southern blot analysis. As shown in Supplemental Figure S1, a 2.9-kb hybridised band with the CAT probe was present in the transformed microalgae, whereas this band was not present in the wild type, demonstrating the integration of the CAT gene in the plastid genome. We observed that even the wild-type control showed two hybridised bands, which could be due to the similarity between the genomic sequences and the probe sequences used.

### Transcript Levels of the *CAT* and *eGFP* Reporter Genes Quantified by Real-Time PCR

**Fig. 4:**
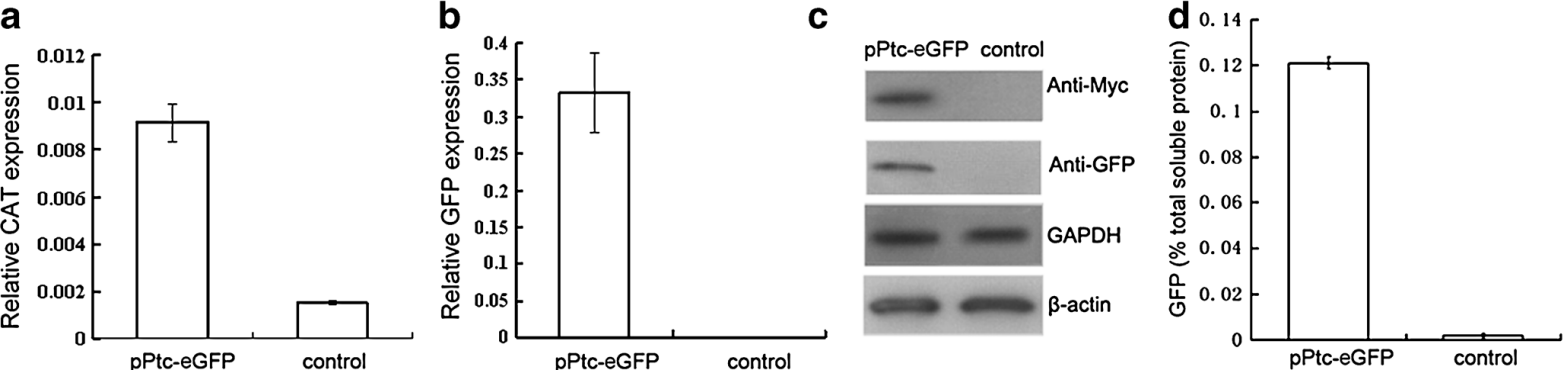
mRNA and protein expression of the reporter genes determined by qPCR, Western blot and ELISA analysis*.* pPtc-eGFP: transformed *P. tricornutum* and control: untransformed *P. tricornutum*. **a** qPCR of CAT expression. **b** qPCR of eGFP expression. **c** Western blot of eGFP expression. The proteins (20 μg) were separated on 12 % SDS–PAGE gels, blotted to PVDF membranes and probed with antibodies. Anti-Myc and anti-GFP antibodies were used for eGFP detection; GAPDH and β-actin were used as internal controls. **d** eGFP accumulation was determined by ELISA

The relative transcript levels of the *CAT* (Fig. 4a) and *eGFP* (Fig. 4b) target genes compared with the control were quantified after normalisation to *β-actin*, and both of the reporters showed relatively high expression. Moreover, the transcript abundance of *eGFP* was even higher, i.e., 33.34-fold, compared with *CAT*. This could be due to the advantages of the endogenous promoter for *eGFP* over the bacterial promoter used in the *CAT* cassette.

### Western Blot and ELISA Analyses of the eGFP Reporter

The expression of the *eGFP* gene in the transformed cells was confirmed by Western blot analysis (Fig. 4c). Anti-Myc and anti-GFP antibodies were used as the probe for the Western blots, which showed a specific cross-reacting band with a molecular mass of 28 kDa in the transformed cells, corresponding to the predicted size of eGFP, while there was no signal in the untransformed cells. This result confirmed the successful expression of the eGFP protein in the transformed cells using the plastid expression system in addition to demonstrating the feasibility of detecting recombinant fusion proteins by a Myc-tag.

To determine the quantity of the eGFP protein, the transformed microalgal cells were analysed by ELISA. Using an eGFP standard, the recombinant protein was shown to accumulate at a level up to 0.121 % of the total soluble protein in the transformed microalgae (Fig. 4d).

### Plastid Localisation of eGFP in Transformed Microalgae

**Fig. 5:**
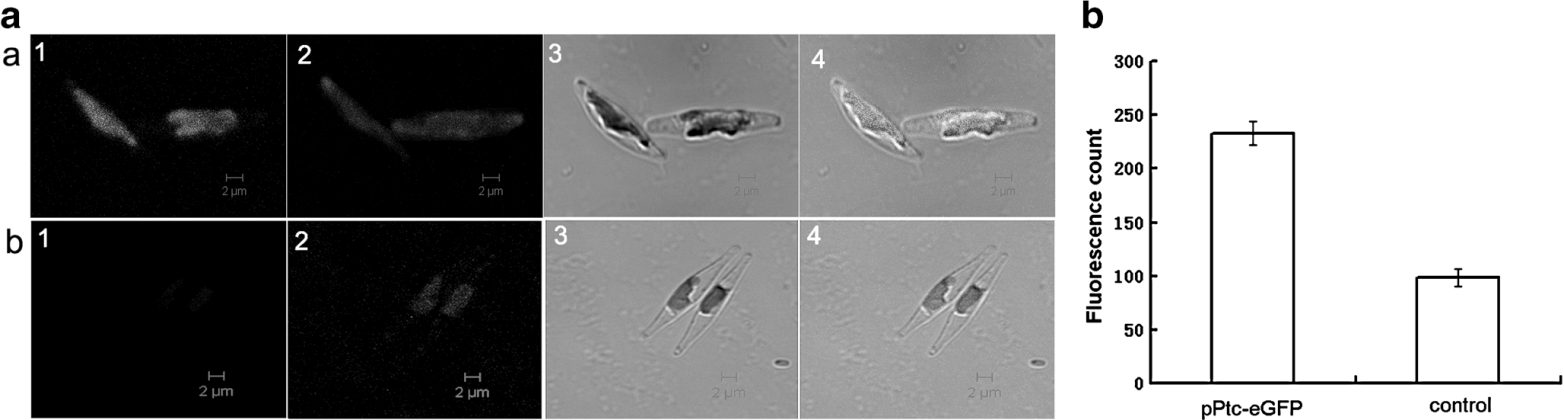
eGFP expression in plastid-transformed *P. tricornutum* analysed by confocal microscopy and flow cytometry. **a** Confocal images showing eGFP expression. *a* pPtc-eGFP transformed *P. tricornutum*, and *b* untransformed *P. tricornutum. 1* Green fluorescence of eGFP, *2* red fluorescence of the plastids, *3* DIC (differential interference contrast) and *4* overlay image indicating the co-localisation of *green and red fluorescence. Bars* = 2 μm. **b** The GFP fluorescence of triplicate samples was determined by flow cytometry, and their relative fluorescence intensities were calculated by subtracting the background counts. The *left and right columns* represent the control and pPtc-eGFP transformed cells, respectively. The images were taken with the same gain settings of the microscope

The fluorescence of pPtc-eGFP transformed microalgae cells was examined using confocal microscopy. Strong green fluorescence was observed in the transformed cells in the green channel (Fig. 5a, a-1), while no fluorescence was detected in the untransformed cells (Fig. 5a, b-1). Moreover, the fluorescence was exclusively restricted to the plastids (Fig. 5a, a-4), which was indicated by the co-localisation of eGFP and autofluorescence of plastid due to chlorophyll. This result further confirmed the plastid-specific expression of the heterologous gene cloned in the newly derived vector pPtc-T. There was also a difference in cell morphology. The eGFP-expressing cells were thicker and shorter when compared to the control, whereas the control cells were long and spindle-shaped (Fig. 5a, b). The results from the cytometry analysis showed high GFP fluorescence intensity in the eGFP-expressing cells, while wild-type cells showed only trace fluorescence caused by the overlap of emission spectra between GFP and algal autofluorescence (Fig. 5b). These results demonstrate that the eGFP reporter gene was successfully expressed at relatively high levels in the plastids. The growth speeds of the transformed and untransformed microalgae cultured in the liquid medium without chloramphenicol showed no visible difference, suggesting that the diatoms could tolerate the higher production of plastid encoded proteins.

## Discussion

Heterologous gene expression in the nucleus in some of the microalgae species has been achieved with relatively low expression levels. It is imperative to develop a commercial-scale microalgae bioreactor. Our study clearly demonstrates stable gene expression in the plastids of the diatom *P. tricornutum*, which could be a proof-of-principle plastid gene expression system for diatoms and an interesting tool to study diatom plastids.

This is the first plastid genetic transformation reported for the diatom based on an electroporation gene delivery mechanism, which is an economical and simple method. Electroporation has been widely used for the delivery of the DNA into microbes, plant cells and animal cells, but so far, there are only few reports in algae. Green algae species that have been successfully transformed by electroporation include *C. reinhardtii* (Brown et al. 1991) and *Scenedesmus obliquus* (Guo et al. 2013), with an efficiency of approximately 0.001 and 0.0005 %, respectively. To date, plastid transformation has solely relied on microprojectile bombardment using the Biolistic PDS-1000/He Particle Delivery System (Bio-Rad, USA). Plastid transformation in red alga *Porphyridium* species was also achieved using a gene gun system (Lapidot et al. 2002). Microprojectile bombardment is an expensive method in terms of both instrumentation and running cost, which limits its use in many laboratories. Our study using the electroporation technique could provide a rather low-cost and feasible way to transform microalgal plastids compared to the routine biolistic approach. The transformation efficiency was calculated to be 0.1 % (5 × 10^3^ transformants in 5 × 10^6^ cells per 0.4 μg DNA), which is relatively higher than that reported for the red microcroalga *Porphyridium* sp., which was up to 2.5 × 10^−4^ per μg DNA (Lapidot et al. 2002). Moreover, the high transformation efficiency makes this approach a highly promising tool for the genetic engineering of microalgae. Considering the possible presence of episomal plasmids in certain microalgae cells during the initial screening and subculture, it is necessary to examine the transformed microalgae using molecular approaches, such as Southern blotting, Northern blotting or Western blotting. The maintenance of the transplastomic lines can be achieved through long-term subculture without applying a selective pressure (Coll 2006).

A high-efficiency plastid transformation system was developed in this study, and this may be attributed to the selection of the specific homologous recombination sequences and also due to the presence of unique IR regions in the *P. tricornutum* plastid genome. The transgene is targeted to the particular IR, followed by the phenomenon of copy correction, which duplicates the introduced transgene into the other IR as well. This process causes transgenes to stably integrate at several sites within the plastid genome. Compared with transcriptionally silent spacer regions, transcriptionally active spacer regions offer unique advantages, such as the insertion of transgenes without 5′ or 3′ untranslated regions (UTRs) or promoters. The transcriptionally active intergenic region between the *trnI*-*trnA* genes within the rrn operon, existing in the IR region of the plastid genome, has been used for integration. High levels of expression of heterologous genes integrated at this site have been reported (Cosa et al. 2001). Plastid vectors may also carry an origin of replication that facilitates replication of the plasmid inside the plastid, thereby increasing the template copy number for homologous recombination and enhancing the probability of transgene integration (Verma and Daniell 2007). Moreover, the CAT reporter gene expressed in transformed *P. tricornutum* was sufficient to confer resistance to chloramphenicol, which contributed to the effective selection of the transgenic microalgae.

Generally, the progress of biotechnology/genetic engineering in microalgae has been slow when compared to other systems. In the case of *P. tricornutum*, the transformation efficiencies reported to date were even lower than those for other pennate diatoms, such as *Cylindrotheca fusiformis*, and the centric diatom *Thalassiosira pseudonana* (Poulsen et al. 2006; Poulsen and Kröger 2005). In this study, we used the *rbcL* promoter from the *P. tricornutum* plastid genome. The *rbcL* promoter is considered to be a strong and constitutive promoter, and it has been widely used in plastid expression vector systems (Verma and Daniell 2007). Additionally, a new approach was employed for easy cloning of the gene of interest between the promoter and terminator in the plasmid pPtc-T when compared to the other commonly used vectors. The two specially designed adjacent *Xcm*I sites created an extra “T” at both 3′-ends of the vector upon *Xcm*I digestion, which allows for the efficient cloning of any gene into the *P. tricornutum* plastid via the TA system. The Myc-tag in the vector provides a tool for testing and purifying the recombinant fusion protein with commercially available reagents to study the heterologous genes expressed in the *P. tricornutum* plastid. Our study shows that the expression level of eGFP was relatively high in the transformed *P. tricornutum* compared with the plastid expression in *C. reinhardtii*, where eGFP without codon optimisation accumulated ~0.006 % of total soluble protein (Franklin et al. 2002). Considering the profound effect of codon usage on the expression of heterologous proteins in microalgae plastids (Mayfield et al. 2007), the current *P. tricornutum* plastid expression system would be expected to have the potential of a much higher protein yield for codon-optimised genes. Interestingly, the morphology of the transformed cells became egg-shaped from the original fusiform, and the size was also smaller than that of the untransformed wild-type cells. Genetic engineering of *P. tricornutum* will be particularly useful for the industrial production of value-added proteins and also to improve its nutritional value, as it is widely used as the live feed in the aquaculture industry.

## Supplemental Figure

Below is the link to the electronic supplementary material.
High resolution image (TIFF 1259 kb)
