# Supplementary figures and images for: Construction of Novel Chloroplast Expression Vector and Development of an Efficient Transformation System for the Diatom Phaeodactylum tricornutum
Source: Mar Biotechnol (NY). 2014 Apr 26;16(5):538–46. doi: 10.1007/s10126-014-9570-3 (PMC4169106; doi:10.1007/s10126-014-9570-3)

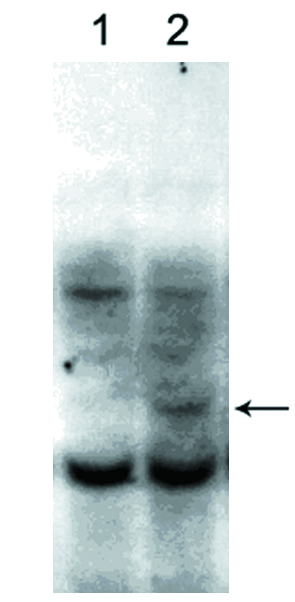

Supplement: Supplementary file 1 — High resolution image (TIFF 1259 kb) [file 10126_2014_9570_MOESM1_ESM.tif]
